# Safety and efficacy of 48-week pegylated interferon-*α*-2b therapy in patients with hepatitis B virus-related compensated liver cirrhosis: a pilot observational study

**DOI:** 10.3389/fmed.2024.1489671

**Published:** 2024-12-04

**Authors:** Zehong Wang, Xuanxuan Wang, Li Zhou, Shaoyuan Shi, Yongli Hua, Yinong Feng

**Affiliations:** Department of Hepatology, The Third People’s Hospital of Taiyuan, Taiyuan, Shanxi Province, China

**Keywords:** pegylated interferon-**α**, hepatitis B virus, liver cirrhosis, antiviral therapy, clinical cure

## Abstract

**Background:**

Pegylated interferon-*α* (PEG-IFN-α) therapy could decrease hepatitis B surface antigen (HBsAg) and improve long-term prognosis of hepatitis B virus (HBV) infection. However, studies on safety and efficacy of PEG-IFN-*α* for patients with HBV-related cirrhosis are limited.

**Methods:**

This was a single-center study. Fifty-four patients with HBV-related compensated cirrhosis were enrolled. All patients received subcutaneous injection of PEG-IFN-*α*-2b 180 μg per week for 48 weeks. The monotherapy of PEG-IFN-α-2b was used for treatment-naïve patients, while addition of PEG-IFN-α-2b to on-going nucleos(t)ide analogs (NAs) was used for NAs-experienced patients. Clinical symptoms, laboratory tests, examination indicators, and adverse events were collected at each observational time point.

**Results:**

Forty-two patients achieved undetectable serum HBV DNA at 48 weeks post-therapy. HBsAg level was significantly reduced at 48 weeks post-therapy (227.2 IU/mL vs. 1,668 IU/mL; *p* < 0.001), especially in NAs-experienced patients (161.0 IU/mL vs. 1,207 IU/mL; *p* = 0.005). Three patients achieved HBsAg loss, and two of them obtained HBsAg seroconversion. There were no significant differences in liver stiffness measurement, thickness and length of spleen, or diameter of portal vein between baseline and 48 weeks post-therapy (*p* > 0.05). The aminotransferase levels were increased, while white blood cells, neutrophils, and platelets counts were decreased during PEG-IFN-*α*-2b therapy (*p* < 0.05), especially in treatment-naïve patients. Three patients discontinued PEG-IFN-α-2b therapy due to severe adverse events. No patients suffered with virological breakthrough or progressed to end-stage liver diseases during observational period.

**Conclusion:**

A finite course of PEG-IFN-*α*-2b therapy was well-tolerated, and reduced HBsAg level without accelerating disease progression in patients with HBV-related compensated cirrhosis.

**Clinical trial registration:**

This trial is a part of ZhuFeng Project (ClinicalTrials.gov, identifier NCT04035837).

## Introduction

The World Health Organization estimated that 257 million people were living with chronic hepatitis B virus (HBV) infection in 2015, leading to 0.88 million deaths every year worldwide due to HBV-related end stage liver diseases, such as decompensated cirrhosis, liver failure, and hepatocellular carcinoma (HCC) (1, 2). China has changed from a highly endemic to an intermediate endemic area for HBV infection in the past three decades (3, 4). Hepatitis B surface antigen (HBsAg) prevalence among people aged 1 ~ 29 years declined 46% during 1992 to 2006 (from 10.1 to 5.5%) (5) and 52% during 2006 to 2014 (from 5.5 to 2.6%) (4). There are approximate 70 million HBsAg carriers with 5 ~ 6% prevalence at present in China (1). Thus, China still has the world’s largest burden of HBV infection and will be the major contributor toward the global elimination of hepatitis B by 2030 (1, 6).

Effective inhibition of HBV replication by antiviral therapy delays or prevents the progression from HBV-related compensated cirrhosis to decompensated cirrhosis, leading to the further reduction in the risk of incidence of HBV-related liver failure and HCC (7). Moreover, HBsAg loss or seroconversion, which is an indicator of functional cure, reduces the occurrence of liver cirrhosis and HCC (8) and improves the long-term outcomes than those who remain HBsAg positive (9–11). Currently, there are two main therapeutic recommendations for chronic hepatitis B (CHB) patients: treatment with nucleos(t)ide analogs (NAs) [including entecavir (ETV), tenofovir (TDF), tenofovir alafenamide (TAF), and tenofovir amibufenamide (TMF)] or pegylated interferon-*α* (PEG-IFN-α) (12–15). Although NAs could effectively inhibit HBV replication, the cumulative rate of HBsAg loss was low, which is even comparable with that of untreated patients (16). PEG-IFN-α reveals both antiviral and immunomodulatory activity with sustained response after a finite course of therapy, resulting in a higher rate of both HBsAg loss and HBsAg seroconversion (17, 18). Patients with lower baseline HBsAg and HBV DNA level (19), rapid reduction of HBsAg during treatment (20), as well as the CC and TT genetic polymorphisms of interleukin-28B (21) are more likely to respond effectively to PEG-IFN-*α* therapy.

For hepatitis B e antigen (HBeAg)-positive CHB patients who had achieved HBV DNA inhibition and low HBeAg level in response to ETV therapy, switching to a finite course (48 weeks) of PEG-IFN-α-2a robustly elevated the rates of HBeAg seroconversion and HBsAg clearance (“OSST trial”) (22). Moreover, HBeAg seroconversion and HBsAg loss were sustained in most patients during off-treated 1 year follow-up (23). For CHB patients who achieved virological response and HBeAg loss to a previous NA treatment, switching to PEG-IFN-*α*-2a led to high rate of HBsAg loss at 48 weeks (14.4%) and 96 weeks (20.7%) post PEG-IFN-α-2a therapy (“New Switch study”) (24). For HBeAg-positive CHB patients, PEG-IFN-*α*-2a add-on to on-going ETV strategy led to significantly more decline in HBV DNA, HBsAg, and HBeAg level and higher proportion of HBeAg loss (25). These studies were mainly focused on sustained virological and serological response to PEG-IFN-*α* therapy in CHB patients. Several previous reports also showed that PEG-IFN-α was also effective and safe for patients with HBV-related fibrosis (26, 27). However, the application of PEG-IFN-α in patients with HBV-related compensated liver cirrhosis remains to be explored.

HBsAg seroclerance decreased the risk of hepatic decompensation in CHB patients (28), and was associated with a lower risk of late recurrence of HBV-related HCC (29). Cost-effectiveness analyses also revealed that earlier implementation of expanded antiviral therapy could decrease HBV-related complications and deaths in compensated cirrhosis patients with low-level viremia and even in untreated minimally active CHB patients, which contributed positively to individual clinical benefits and national healthcare budgets (30–32). Thus, although the medicine expense for PEG-IFN-*α* is higher than NA treatment, it could not only strongly inhibits viral replication and reduces HBsAg level, but also eliminates progression to end-stage liver diseases, leading to the down-regulation of both direct and indirect costs as well as potential long-term benefits. Furthermore, the immunomodulatory property of PEG-IFN-*α* could enhance natural killer cell activation (33, 34) and restore viral specific CD8^+^ T cell response (35), revealing particularly advantageous in cirrhotic patients.

Therefore, we conducted a real-world observational prospective study to investigate the safety and efficacy of PEG-IFN-*α* therapy for patients with HBV-related compensated cirrhosis.

## Methods

### Ethics statement

The study protocol was approved by the Institutional Review Board of The Third People’s Hospital of Taiyuan on August 11st, 2022 (Approval No. 2022–09). Written consent was obtained from all enrolled patients, whose data were anonymized for all analyses. This study involving human participants was in accordance with the ethical standards of the institutional and national research committee and with the 1964 Helsinki Declaration and its later amendments or comparable ethical standards. This trial is a part of ZhuFeng Project (The Clinical Cure Project of Chronic Hepatitis B in China; ClinicalTrials.gov ID: NCT04035837).

### Enrollments of study patients

**Figure 1:**
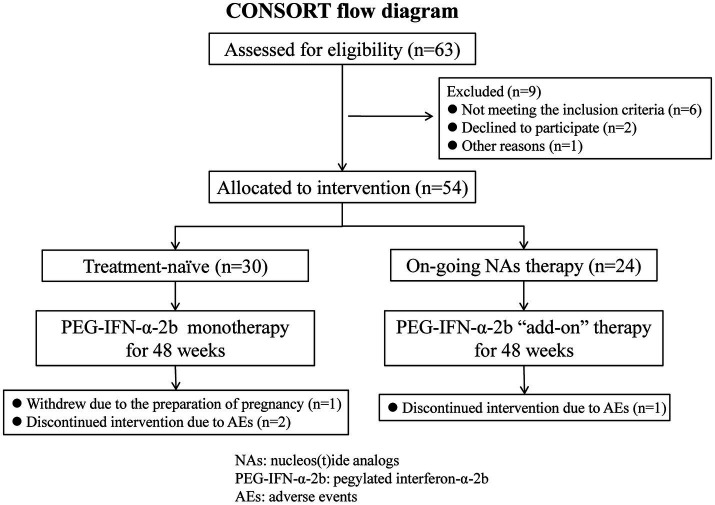
Consolidation standards of reporting trails (CONSORT) flow diagram for this study.

This was a single-center study, which was conducted at the Department of Hepatology of The Third People’s Hospital of Taiyuan between September 2022 and July 2024. The study met the Consolidation of Standards of Reporting Trails (CONSORT) reporting standards, and the CONSORT flow diagram is shown in Figure 1. The diagnosis of HBV-related compensated liver cirrhosis was in accordance with the Chinese Guidelines on the Management of Liver Cirrhosis (36): ① HBsAg was positive for more than 6 months; ② Imaging assessments, such as sonography, computed tomography (CT), and magnetic resonance imaging (MRI) scan, revealed cirrhosis (morphological changes of the liver, formation of liver nodules, and portal hypertension); ③ Liver stiffness measurement (LSM) result complied with the diagnostic cutoff of cirrhosis; ④ The patients did not suffer with the evidence of decompensated complications, including ascites, gastroesophageal varices hemorrhage, sepsis, hepatic encephalopathy, and hepatorenal syndrome. The exclusive criteria included: ① Peripheral blood neutrophils count <1.25 × 10^9^/L or platelet count <75 × 10^9^/L; ② Alanine aminotransferase (ALT) level higher than five times of the upper limit of normal (ULN); ③ Co-infected with other hepatitis virus (hepatitis A, C, D, or E virus) or human immunodeficiency virus; ④ Afflicted with other liver diseases, such as autoimmune liver disease, alcoholic liver disease, alcoholic liver disease, or Wilson’s disease; ⑤ Afflicted with hyperthyroidism or hypothyroidism; ⑥ Afflicted with solid cancers or leukemia; ⑦ Receiving chemotherapy or immunosuppressive treatments; ⑧ Afflicted with important organ failure; ⑨ Pregnant or lactating women.

### Study design

This was a single center, prospective, observational study. All enrolled patients received PEG-IFN-*α*-2b (Y shape, 40 kD; 180 μg, subcutaneous injection weekly; Xiamen Amoytop Biotech Co., Ltd., Xiamen, Fujian Province, China) therapy. The monotherapy of PEG-IFN-*α*-2b was used for treatment-naïve patients, while the “add-on” strategy, which defined as addition of PEG-IFN to on-going NAs (37), was used for patients with on-going NAs regimen. The dosage of PEG-IFN-*α*-2b was adjusted to 135 μg per week if the neutrophils count ≤0.75 × 10^9^/L or platelets count <50 × 10^9^/L, while PEG-IFN-*α*-2b was discontinued if the neutrophils count ≤0.50 × 10^9^/L or platelets count <25 × 10^9^/L or serious adverse events occurred according to the instruction of the manufacturer. The total observation duration of PEG-IFN-α-2b therapy was 48 weeks. The four observation time point was baseline, 12 weeks, 24 weeks, and 48 weeks post PEG-IFN-α-2b therapy.

### Virological, biochemical, serological, and imaging assessments

Laboratory results including white blood cells (WBC), neutrophils, hemoglobin (HGB), platelets, ALT, aspartate aminotransferase (AST), albumin, HBV DNA, HBsAg, anti-HBs, HBeAg, anti-HBe, anti-HBc in the serum were measured at each observational time point from all enrolled patients. Serum HBV DNA was quantified by real-time fluorescence quantitative polymerase chain reaction using a commercial HBV DNA detection kit (Xiamen Amplly, Xiamen, Fujian Province, China) with the detection limit of 50 IU/mL. HBsAg, anti-HBs, HBeAg, anti-HBe, and anti-HBc was quantified using the ARCHITECH HBsAg, anti-HBs, HBeAg, anti-HBe, and anti-HBc reagent kit (Abbott GmbH & Co., KG., Wiesbaden, Germany), respectively. The detection limit for HBsAg was 0.05 IU/mL, and anti-HBs level higher than 10 IU/mL was considered as positive. HBsAg loss was defined as the HBsAg level less than 0.05 IU/mL. The length and thickness of spleen, as well as diameter of portal vein was assessed by sonography, and LSM was performed using transient elastography (FibroScan, EchoSens, Paris, France) at baseline and 48 weeks post PEG-IFN-*α*-2b therapy, respectively. The sonography and Fibroscan test was performed by two senior and experienced doctors.

### Statistical analysis

SPSS 23.0 was used for general statistical analysis. Shapiro–Wilk test was used for normal distribution assay of continuous variables. The continuous variables following normal distribution were described as mean ± standard deviation (SD), and the statistical significance was determined by either Student’s *t-*test or one-way analysis of variance followed by Tukey test. The continuous variables following skewed distribution were described as median and interquartile range (Q1, Q3), and the statistical significance was determined by Mann–Whitney *U* test or Kriskal–Wallis *H* test followed by Dunn’s multiple comparison test. Categorical variables were presented as count and percentage, and statistical significance was determined by Chi-squared test or Fisher’s exact test. All *p*-values were two-sided, and type I error was set as 5%.

## Results

### Baseline characteristics of enrolled patients

A total of 54 patients with HBV-related compensated liver cirrhosis were enrolled in this study. The baseline characteristics of for patients were shown in Table 1. Thirty-one (57.41%) patients suffered with splenomegaly and 22 (40.74%) patients had widened portal vein (≥1.3 cm) based on imaging assessments. The mean or median levels corresponding to clinical indices, including liver function and blood routine test, were in the normal ranges. Thirty patients were treatment-naïve, and started PEG-IFN-*α*-2b monotherapy. Other 24 patients had ongoing NAs therapy, (including 11 of ETV, 12 of TAF, and 1 of TMF) and received PEG-IFN-α-2b add-on therapy. Six (20.00%) patients in treatment-naïve group had undetectable serum HBV DNA, but five (20.83%) patients in NAs-experienced group still had detectable serum HBV DNA (Table 2). There were no statistical differences in HBsAg level, HBeAg-positive rate, liver function, or blood routine test indices between treatment-naïve group and NAs-experienced group (all *p* > 0.05, Table 2).

**Table 1:** Baseline characteristics of enrolled patients.

Characteristic	Value
Patients enrolled, *n*	54
Male gender, *n* (%)	33 (61.11%)
Age, years, mean ± SD	43.02 ± 8.40
Course of hepatitis B, years, median (interquartile range)	15.00 (5.50, 21.25)
Treatment-naïve, *n* (%)	30 (55.56%)
NAs-experienced, *n* (%)	24 (44.44%)
ETV-experienced, *n* (%)	11 (20.37%)
TAF-experienced, *n* (%)	12 (22.22%)
TMF-experienced, *n* (%)	1 (1.85%)
HBV DNA undetectable (<50 IU/mL), *n* (%)	25 (46.30%)
HBV DNA detectable (>50 IU/mL), *n* (%)	29 (53.70%)
HBsAg level, IU/ml, median (interquartile range)	1,668 (446.2, 4,842)
HBeAg positive, *n* (%)	9 (16.67%)
Thickness of spleen, cm, mean ± SD	3.83 ± 0.67
Length of spleen, cm, mean ± SD	10.48 ± 1.63
Splenomegaly, *n* (%)	31 (57.41%)
Diameter of portal vein, cm, mean ± SD	1.20 ± 0.15
Diameter of portal vein ≥1.3 cm, *n* (%)	22 (40.74%)
LSM, kPa, median (interquartile range)	7.40 (5.75, 10.90)
ALT, IU/ml, median (interquartile range)	26.00 (20.00, 36.00)
AST, IU/ml, median (interquartile range)	24.50 (21.00, 32.00)
Albumin, g/L, mean ± SD	44.85 ± 5.07
WBC, ×10^9^/L, mean ± SD	5.41 ± 1.35
Neutrophils, ×10^9^/L, mean ± SD	3.11 ± 1.08
Platelets, ×10^9^/L, mean ± SD	176.3 ± 54.15
HGB, g/L, mean ± SD	144.8 ± 20.16

SD, standard deviation; NAs, nucleos(t)ide analogs; ETV, entecavir; TAF, tenofovir alafenamide; TMF, tenofovir amibufenamide; HBsAg, hepatitis B surface antigen; HBeAg, hepatitis B e antigen; LSM, liver stiffness measurement; ALT, alanine aminotransferase; AST, aspartate aminotransferase; WBC, white blood cells; HGB, hemoglobin.

**Table 2:** Baseline characteristics of treatment-naïve group and NAs-experienced group.

Characteristic	Treatment-naïve group	NAs-experienced group	*p*-value
Patients enrolled, *n*	30	24	–
Male gender, *n* (%)	15 (50.00%)	18 (33.33%)	0.061
Age, years, mean ± SD	42.73 ± 9.07	43.38 ± 7.60	0.783
Course of hepatitis B, years, median (interquartile range)	15.00 (11.50, 21.25)	15.00 (2.25, 22.50)	0.413
HBV DNA undetectable (<50 IU/mL), *n* (%)	6 (20.00%)	19 (79.17%)	<0.001
HBV DNA detectable (>50 IU/mL), *n* (%)	24 (80.00%)	5 (20.83%)	<0.001
HBsAg level, IU/ml, median (interquartile range)	2,588 (419.5, 5,312)	1,207 (514.9, 2,910)	0.220
HBeAg positive, *n* (%)	4 (13.33%)	5 (20.83%)	0.462
Thickness of spleen, cm, mean ± SD	3.69 ± 0.66	4.00 ± 0.66	0.089
Length of spleen, cm, mean ± SD	10.29 ± 1.62	10.71 ± 1.65	0.353
Splenomegaly, *n* (%)	15 (50.00%)	16 (66.67%)	0.218
Diameter of portal vein, cm, mean ± SD	1.17 ± 0.14	1.24 ± 0.15	0.102
Diameter of portal vein ≥1.3 cm, *n* (%)	9 (30.00%)	13 (54.17%)	0.073
LSM, kPa, median (interquartile range)	7.05 (5.75, 10.70)	8.50 (5.68, 11.95)	0.444
ALT, IU/ml, median (interquartile range)	28.50 (18.00, 43.50)	24.50 (21.25, 34.25)	0.862
AST, IU/ml, median (interquartile range)	25.50 (20.75, 35.00)	24.50 (21.25, 30.75)	0.502
Albumin, g/L, mean ± SD	44.23 ± 3.05	45.63 ± 6.81	0.321
WBC, ×10^9^/L, mean ± SD	5.36 ± 1.10	5.46 ± 1.62	0.784
Neutrophils, ×10^9^/L, mean ± SD	140.6 ± 20.75	149.9 ± 18.54	0.093
Platelets, ×10^9^/L, mean ± SD	181.4 ± 48.76	169.9 ± 60.68	0.445
HGB, g/L, mean ± SD	3.10 ± 0.82	3.11 ± 1.39	0.981

NAs, nucleos(t)ide analogs; SD, standard deviation; ETV, entecavir; TAF, tenofovir alafenamide; TMF, tenofovir amibufenamide; HBsAg, hepatitis B surface antigen; HBeAg, hepatitis B e antigen; LSM, liver stiffness measurement; ALT, alanine aminotransferase; AST, aspartate aminotransferase; WBC, white blood cells; HGB, hemoglobin.

### PEG-IFN-*α*-2b therapy reduced the HBsAg level without accelerating disease progression in patients with HBV-related liver cirrhosis

**Figure 2:**
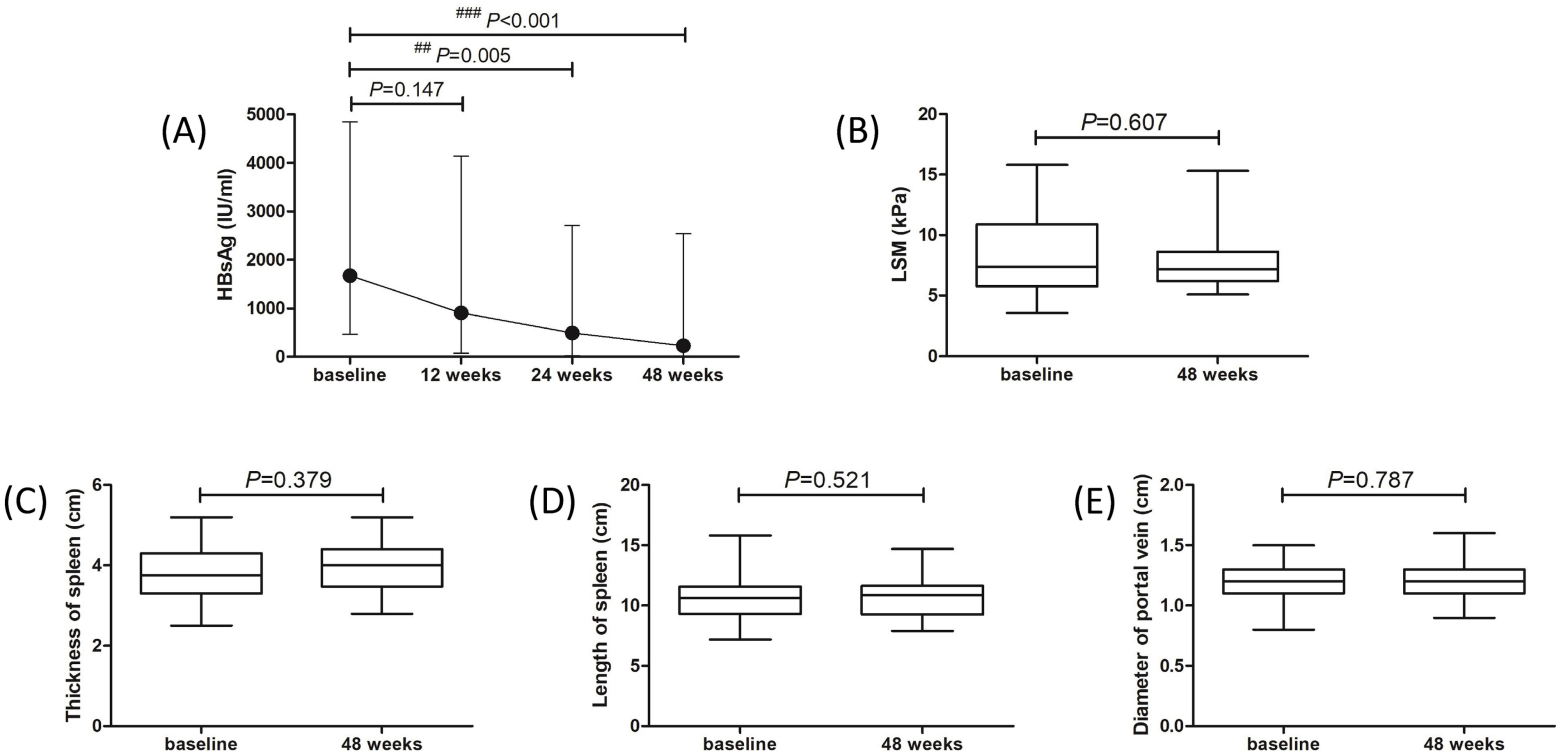
Evolution of therapeutic response to pegylated interferon-*α*-2b over 48 weeks in all enrolled patients with hepatitis B virus-related compensated liver cirrhosis. **(A)** Changes of serum HBsAg level at different observational time points. The points indicate median, while the bars indicate interquartile range. Statistical analyses were performed using Kriskal-Wallis *H* test followed by Dunn’s multiple comparison test. **(B)** Change of liver stiffness measurement (LSM) at 48 weeks compared with baseline. **(C)** Change of thickness of spleen at 48 weeks compared with baseline. **(D)** Change of length of spleen at 48 weeks compared with baseline. **(E)** Change of diameter of portal vein at 48 weeks compared with baseline. The central lines indicate median or mean, the boxes indicate standard deviation or interquartile range, and the bars indicate the minimum to maximum range. Statistical analyses were performed using Mann–Whitney *U* test or Student’s *t*-test.

Fifty patients (28 of treatment-naïve patients and 22 of NAs-experienced patients) completed the 48-week PEG-IFN-*α*-2b-based therapy. One patient withdrew the informed consent due to the preparation of pregnancy at 20 weeks post PEG-IFN-α-2b monotherapy. Two patients discontinued PEG-IFN-α-2b therapy due to severe hyperthyroidism and continuous thrombocytopenia (<40 × 10^9^/L) at 36 weeks post therapy. One patient who received PEG-IFN-α-2b plus TAF therapy suffered with ascites at 40 weeks post therapy, and discontinued PEG-IFN-α-2b therapy based on the consultation of supervising doctors. At 48 weeks post therapy, 42 (77.78%) patients (22 of treatment-naïve patients and 20 of NAs-experienced patients) achieved virological response with undetectable serum HBV DNA. Three of the nine HBeAg-positive patients had HBeAg loss at 48 weeks post therapy. Three patients achieved HBsAg loss at 48 weeks post therapy, while two of them achieved HBsAg seroconversion. Importantly, the median HBsAg level was gradually reduced at each time point. The HBsAg level at 12 weeks post therapy was 906.3 (73.97, 4,134) IU/ml, which was lower than the baseline level [1,668 (446.2, 4,842) IU/ml], but this difference failed to obtain statistical significance (*p* = 0.147, Figure 2A). The HBsAg levels at 24 weeks and 48 weeks post therapy were 487.7 (23.07, 2,702) IU/ml and 227.2 (12.36, 2,535) IU/ml, respectively, which were robustly lower than the baseline level (all *p* < 0.01, Figure 2A). There were no remarkable differences in LSM (Figure 2B), thickness and length of spleen (Figures 2C,D), or diameter of portal vein (Figure 2E) between baseline and 48 weeks post therapy (all *p* > 0.05).

**Figure 3:**
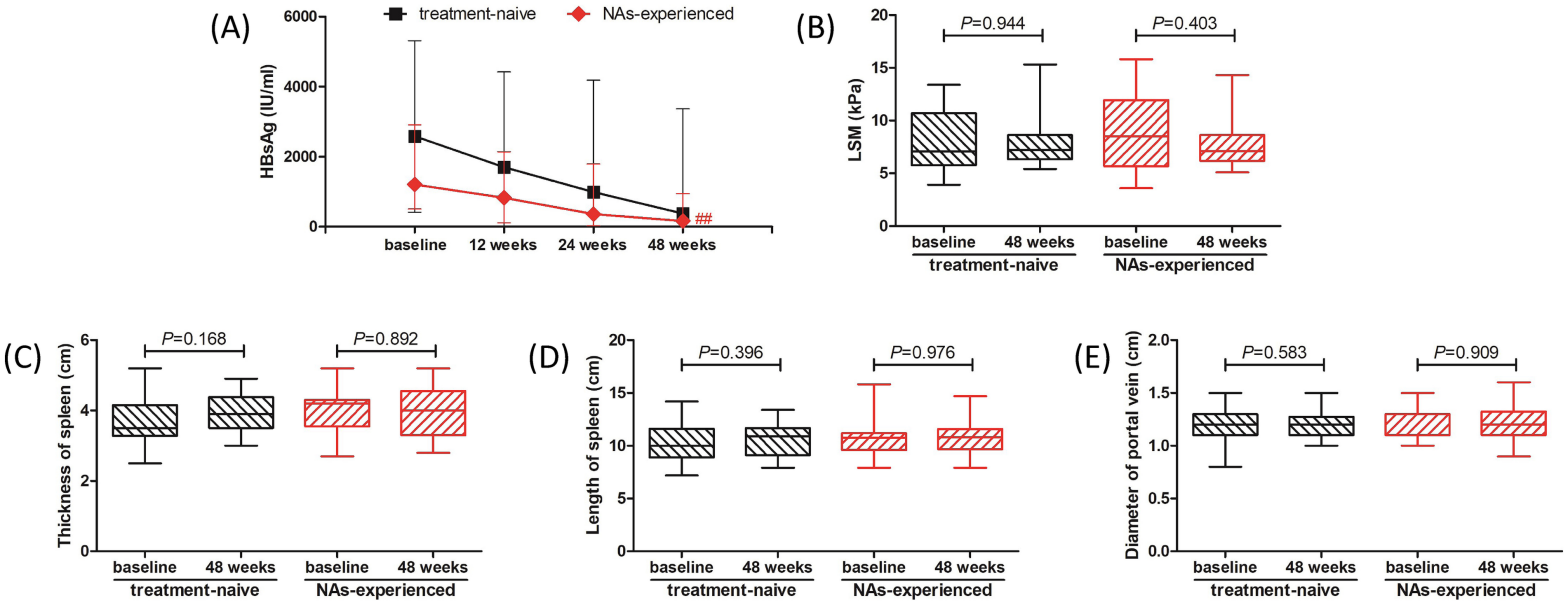
Evolution of therapeutic response to pegylated interferon-α-2b over 48 weeks in treatment-naïve group and nucleos(t)ide analogs (NAs)-experienced group. **(A)** Changes of serum HBsAg level at different observational time points in two groups. The black line indicates treatment-naïve group, while the red line indicates NAs-experienced group. The points indicate median, while the bars indicate interquartile range. Statistical analyses were performed using Kriskal-Wallis *H* test followed by Dunn’s multiple comparison test. **(B)** Change of liver stiffness measurement (LSM) at 48 weeks compared with baseline in two groups. **(C)** Change of thickness of spleen at 48 weeks compared with baseline in two groups. **(D)** Change of length of spleen at 48 weeks compared with baseline in two groups. **(E)** Change of diameter of portal vein at 48 weeks compared with baseline in two groups. The central lines indicate median or mean, the boxes indicate standard deviation or interquartile range, and the bars indicate the minimum to maximum range. Statistical analyses were performed using Mann–Whitney *U* test or Student’s *t* test. ^##^ *p* < 0.01 compared with baseline.

We then analyzed the therapeutic response to PEG-IFN-*α*-2b in treatment-naïve group and NAs-experienced group, respectively. Although there were reduced trends in HBsAg level in both groups, the difference of HBsAg level in treatment-naïve group among different observational time points just missed the statistical significance (*p* = 0.089, Figure 3A, black line). The HBsAg level in NAs-experienced group was remarkably down-regulated at 48 weeks post therapy compared with baseline [161.0 (31.82, 944.0) IU/ml vs. 1,207 (514.9, 2,910) IU/ml; *p* = 0.005, Figure 3A, red line]. There were also no significant differences in LSM (Figure 3B), thickness and length of spleen (Figures 3C,D), or diameter of portal vein (Figure 3E) between baseline and 48 weeks post therapy in either treatment-naïve group or NAs-experienced group (all *p* > 0.05).

### Safety

**Figure 4:**
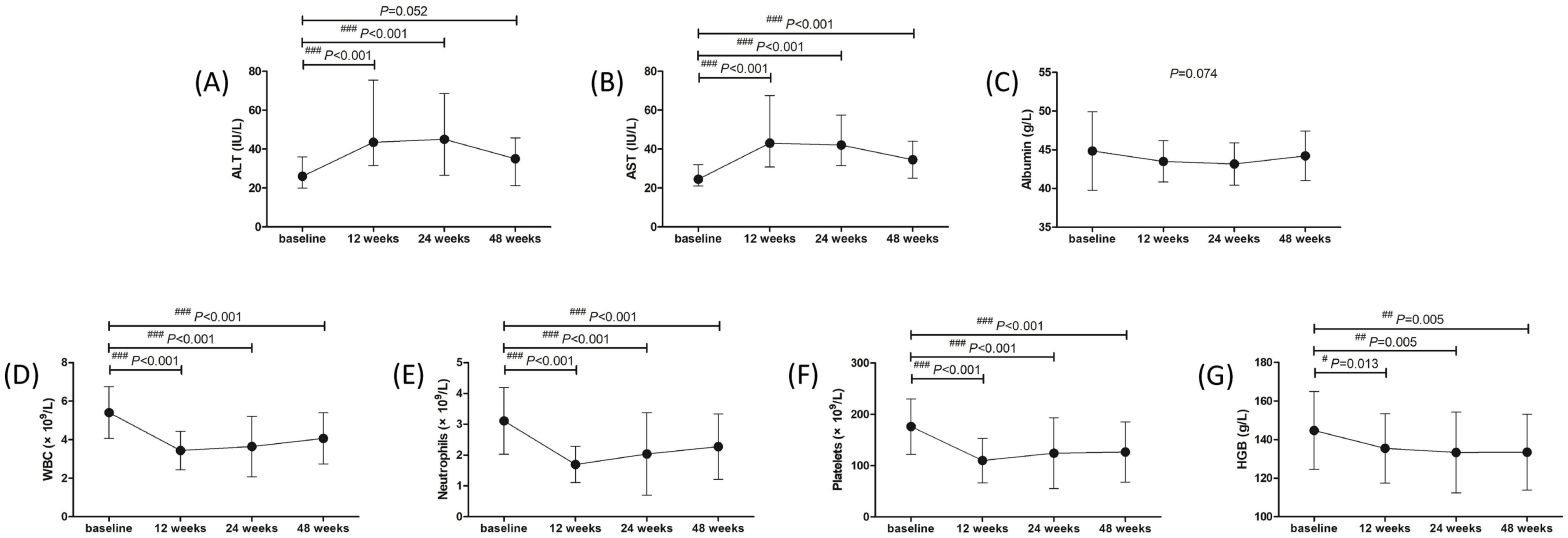
Evolution of liver function and blood routine test in response to pegylated interferon-α-2b over 48 weeks in all enrolled patients with hepatitis B virus-related compensated liver cirrhosis. Changes of **(A)** alanine aminotransferase (ALT) level, **(B)** aspartate aminotransferase (AST) level, **(C)** albumin level, **(D)** white blood cells (WBC) count, **(E)** neutrophils count, **(F)** platelets count, **(G)** hemoglobin (HGB) level at different observational time points. The points indicate median or mean, while the bars indicate interquartile range or standard deviation. Statistical analyses were performed using Kriskal-Wallis *H* test followed by Dunn’s multiple comparison test or one-way analysis of variance followed by Tukey test. ^#^*p* < 0.05, ^##^*p* < 0.01, ^###^*p* < 0.001 compared with baseline.

Adverse events (AEs) were analyzed in all studied population over 48 weeks. The most common symptoms of AEs were fever (53, 98.15%), fatigue (52, 96.30%), weight loss (41, 75.93%), and alopecia (7, 12.96%). Grade 1 ascites was found in one patient. Thyroid dysfunction was found in three (5.56%) patients, and one patient discontinued treatment due to severe hyperthyroidism. ALT and AST levels showed approximate 2-fold elevation at 12 weeks and 24 weeks post therapy (Figures 4A,B). Both ALT and AST levels reduced at 48 weeks post therapy (all *p* < 0.001, Figures 4A,B), but AST level was still higher than the baseline (*p* < 0.001, Figure 4B). ALT flares (>5 × ULN) occurred in four (7.41%) patients, and ALT returned to normal level in response to hepatic protection without discontinued PEG-IFN-*α*-2b treatment. There was no significant difference in albumin level among each observational time point (*p* = 0.074, Figure 4C). WBC and neutrophils count was reduced at 12, 24, and 48 weeks post therapy (all *p* < 0.001, Figures 4D,E), but neutropenia (<1 × 10^9^/L) was only occurred in 11 (20.37%) patients during therapy. Platelets count was also reduced at 12, 24, and 48 weeks post therapy (all *p* < 0.001, Figure 4F). Thrombocytopenia (<75 × 10^9^/L) was occurred in twelve (22.22%) patients during therapy, and one patient discontinued treatment due to severe and continuous thrombocytopenia. Hemoglobin (HGB) level was also reduced in response to PEG-IFN-α-2b therapy (all *p* < 0.05, Figure 4G), but no severe anemia (<60 g/L) was found during therapy. No patients suffered with virological breakthrough or progressed to liver failure or HCC during the observational period.

**Figure 5:**
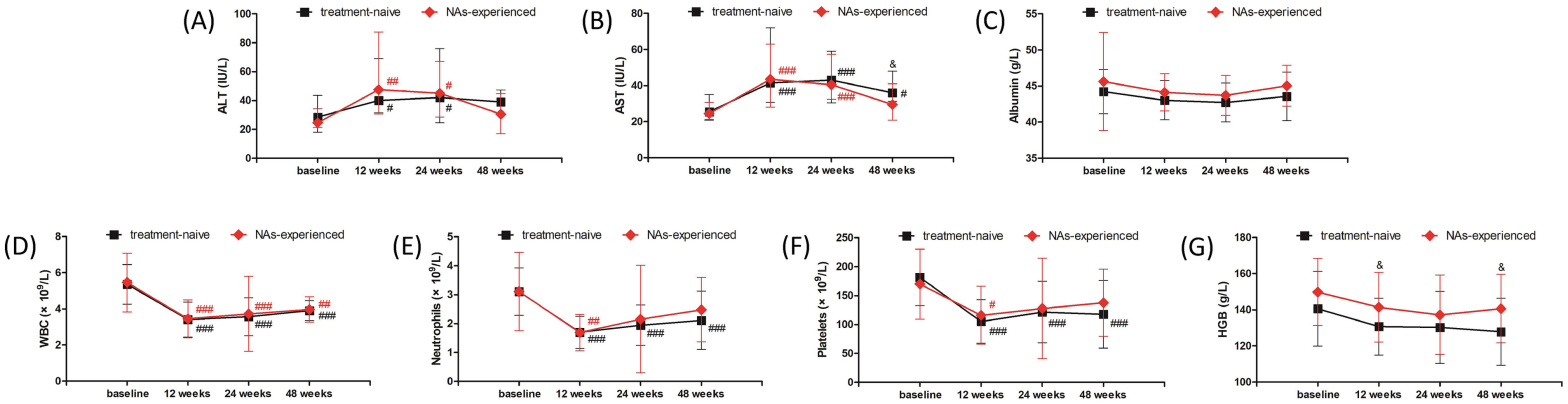
Evolution of liver function and blood routine test in response to pegylated interferon-α-2b over 48 weeks in treatment-naïve group and nucleos(t)ide analogs (NAs)-experienced group. Changes of **(A)** alanine aminotransferase (ALT) level, **(B)** aspartate aminotransferase (AST) level, **(C)** albumin level, **(D)** white blood cells (WBC) count, **(E)** neutrophils count, **(F)** platelets count, **(G)** hemoglobin (HGB) level at different observational time points in two groups. The black line indicates treatment-naïve group, while the red line indicates NAs-experienced group. The points indicate median or mean, while the bars indicate interquartile range or standard deviation. Statistical analyses were performed using Kriskal-Wallis *H* test followed by Dunn’s multiple comparison test or one-way analysis of variance followed by Tukey test. ^#^*p* < 0.05, ^##^*p* < 0.01, ^###^*p* < 0.001 compared with baseline. ^&^*p* < 0.05 compared with NAs-experienced group in the same observational time point.

The safety profile of liver function and blood routine test was also investigated in treatment-naïve group and NAs-experienced group, respectively. The overall trends of liver function and blood routine test was similar in two groups. ALT level was increased at 12 weeks and 24 weeks (all *p* < 0.05), and down-regulated at 48 weeks post therapy (all *p* > 0.05) in both groups (Figure 5A). AST level was also elevated at 12 weeks and 24 weeks post therapy in both groups (all *p* < 0.001, Figure 5B). AST level at 48 weeks post therapy was still higher than baseline in treatment-naïve group (*p* = 0.020, Figure 5B), which was also higher than the level in NAs-experienced group (*p* = 0.029, Figure 5B). There was no significant difference in albumin level among each observational time point in two groups (all *p* > 0.05, Figure 5C). Although WBC count was reduced at 12 weeks, 24 weeks, and 48 weeks post therapy in two groups (all *p* < 0.01, Figure 5D), neutrophils count was returned at 24 weeks and 48 weeks post therapy in NAs-experienced group without statistical significances compared with baseline (all *p* > 0.05, Figure 5E). Similarly, platelets count was also returned at 24 weeks and 48 weeks post therapy in NAs-experienced group without remarkable differences compared with baseline (all *p* > 0.05, Figure 5F). Interestingly, there was no significant difference in HGB level among each observational time points in two groups (all *p* > 0.05, Figure 5G), but HGB level was higher in NAs-experienced group at 12 weeks and 48 weeks post therapy compared with the level in treatment-naïve group at the same observational time point (all *p* < 0.05, Figure 5G).

## Discussion

To the best of our knowledge, this is the first report regarding the efficacy and safety of PEG-IFN-*α*-based therapy to patients with HBV-related liver cirrhosis. All patients confirmed the diagnosis of liver cirrhosis based on the imaging assessments, including the evidence of morphological changes of the liver, formation of liver nodules, and portal hypertension. However, these patients did not suffer with decompensated cirrhosis. PEG-IFN-*α*-2b treatment not only induced the virological response with undetectable serum HBV DNA in most of the patients, but also strongly down-regulated HBsAg levels (the median level reduction from 1,668 IU/mL to 227.2 IU/mL) at 48 weeks post-therapy. Importantly, administration of a finite course of PEG-IFN-*α*-2b did not accelerate the disease progression for cirrhosis, which presented as comparable LSM, thickness and length of spleen, as well as diameter of portal vein post PEG-IFN-*α*-2b therapy. No patients progressed to end-stage liver diseases during 48 weeks of PEG-IFN-α-2b treatment. The overall safety of PEG-IFN-*α*-2b application was good. The most common symptoms of AEs were fever, fatigue, weight loss, and alopecia, which were similar to those in CHB patients and inactive HBsAg carriers during PEG-IFN-*α* treatments (22, 24, 38–40). The changes of liver function and blood routine test were also presented the similar trends to those in chronic HBV infected patients in response to PEG-IFN-*α* therapy (22, 24, 38–40). Only three patients discontinued therapy due to severe AEs, including severe hyperthyroidism, continuous thrombocytopenia, and grade 1 ascites. These severe AEs were recovered after stopping PEG-IFN-*α* therapy and receiving symptomatic treatments. Collectively, 48-week PEG-IFN-α-2b-based therapy was safety, and indicated moderate efficacy for controlling chronic HBV infection in patients with HBV-related compensated liver cirrhosis.

PEG-IFN-α therapy could reach high rate of virological and serological response to patients with chronic HBV infection. The OSST trial revealed 72.0% (59/82) CHB patients achieved undetectable serum HBV DNA (<1,000 copies/ml) after switching from ETV to 48-week PEG-IFN-*α*-2a therapy, while the rate of virological response was 97.8% (90/92) in patients with continuous ETV therapy. Moreover, 8.5% (8/94) CHB patients achieved HBsAg clearance, and 4.3% (4/94) reached HBsAg seroconversion after switching to PEG-IFN-*α*-2a. However, no patients obtained HBsAg loss or seroconversion with continuous ETV treatment (22). The New switch study also demonstrated that switch from NAs to PEG-IFN-α-2a showed sustained HBV DNA inhibition (<200 IU/mL). 14.4% (22/153) patients obtained HBsAg loss at 48 weeks post-PEG-IFN-α-2a therapy, but the serological response rate for HBsAg clearance (20.7%, 31/150) did not robustly increased for a 96-week treatment (24). Wu et al. showed that PEG-IFN-α-2a therapy elevated the HBsAg clearance rate from 1.9% (2/104) in NAs monotherapy group to 37.4% (34/91) in add-on group (38). Wen et al. revealed that more than 50% CHB patients achieved HBsAg loss in both PEG-IFN-*α*-2b monotherapy and NAs add-on PEG-IFN-α-2b group (41). Furthermore, both PEG-IFN-α-2b monotherapy and PEG-IFN-α plus adefovir dipivoxil treatment results in more than 40% of HBsAg loss and more than 30% of HBsAg seroconversion in inactive HBsAg carriers (39, 40, 42, 43). Herein, we showed that 77.78% (42/54) patients with HBV-related compensate cirrhosis had undetectable serum HBV DNA (<50 IU/mL) at 48 weeks post-PEG-IFN-*α*-2b therapy. The rate of virological response was also comparable between treatment-naïve patients (PEG-IFN-α-2b monotherapy, 77.33%, 22/30) and NAs-experienced patients (PEG-IFN-*α*-2b plus NAs therapy, 83.33%, 20/24). This rate was similar to the previous reports regarding to the PEG-IFN-α treatment to HBV DNA suppression in CHB patients (22, 24, 38). We also show that although there were clear trends in HBsAg reduction, only PEG-IFN-α-2b add-on therapy to ongoing NAs could statistically reduce HBsAg level at 48 weeks post therapy. This might partly due to the fact that NAs-experienced patients had lower baseline HBsAg level than treatment-naïve patients, although this difference failed to achieve statistical significance. The limited enrolled patients might also contribute to the certain differences might not reach significance. Moreover, the present data revealed that although HBsAg level was gradually decreased during PEG-IFN-*α*-2b-based therapy, only 5.56% (3/54) patients reached HBsAg loss and 3.70% (2/54) patients obtained HBsAg seroconversion, which were lower than the rates in the published literatures in CHB patients and inactive HBsAg carriers (22, 24, 38–40, 42, 43). The key differences between the current study and other reports were shown in Table 3. In our opinions, the following three reasons might contribute to this difference. Firstly, we used a 48-week finite course of PEG-IFN-*α*-2b because prolonged PEG-IFN-α-2b treatment might induce the incidence of AEs and lead to the acute decompensation in compensated liver cirrhosis. Secondly, baseline HBsAg level less than 1,500 IU/mL was associated with high rate of HBsAg loss in response to PEG-IFN-*α*-2b therapy (22, 24, 38). The median baseline HBsAg level was 1,668 IU/mL, and 53.70% (29/54) patients had baseline HBsAg level higher than 1,500 IU/mL. We also observed that the three patients who achieved HBsAg loss also had moderate low level of baseline HBsAg (14.80 IU/mL, 60.74 IU/mL, and 512.1 IU/mL, respectively). Thirdly, the circulating and hepatic immune environment changed during the progression from CHB to liver cirrhosis (44, 45). The enrichment and elevation of exhausted immune cells in the circulation and liver microenvironment might reduce the responsiveness to PEG-IFN-*α* in HBV-related cirrhotic patients (44, 45).

**Table 3:** The key differences between the current study and other reports.

Study	Enrolled patients	Treatments	Baseline HBV DNA	Baseline HBsAg	Course	HBsAg loss/seroconversion	Ref.
Our study	Decompensated cirrhosis	PEG-IFN-α-2b monotherapy for treatment-naïve and add-on for NAs-experienced	Detectable (> 50 IU/mL) in 53.70% patients	1,668 IU/mL	48 weeks	5.56%/3.70%	
OSST	HBeAg-positive CHB patients, ETV experienced	Switch to PEG-IFN-α-2a monotherapy	≤1,000 copies/ml	3.3 log_10_IU/ml	48 weeks	8.5%/4.3%	(22)
New Switch	HBeAg-negative CHB patients, NAs experienced	Switch to PEG-IFN-α-2a monotherapy	<200 IU/mL	3.2 log_10_IU/ml	48 weeks or 96 weeks	14.4%/13.1% (48 weeks) 20.7%/16.0% (96 weeks)	(24)
Wu et al.	HBeAg-negative CHB patients, NAs experienced	PEG-IFN-α-2a add-on	<100 IU/mL	≤1,500 IU/mL	48 weeks	26.4%/18.7%	(38)
Cao et al.	IHC	PEG-IFN-α-2a monotherapy or PEG-IFN-α-2a combined with adefovir	<2,000 IU/mL	<1,000 IU/mL	48 weeks or 96 weeks	29.8%/20.2% (48 weeks) 44.7%/38.3% (96 weeks)	(39)
Huang et al.	IHC	PEG-IFN-α-2b monotherapy	<2,000 IU/mL	<1,000 IU/mL	48 weeks	84.2%/68.4%	(40)
Wen et al.	IHC and NAs-experienced	PEG-IFN-α-2b monotherapy for IHC; PEG-IFN-α-2a add-on for NAs-experienced	<2,000 IU/mL	<1,000 IU/mL	48 weeks	65.5%/47.3% (IHC) 52.9%/34.3% (NAs-experienced)	(41)
Wu et al.	IHC	PEG-IFN-α-2a or PEG-IFN-α-2b monotherapy	<2,000 IU/mL	<1,500 IU/mL	48 weeks	47.9%/36.6%	(42)
Ning et al.	IHC	PEG-IFN-α-2b monotherapy or with a lead-in period of GM-CSF and vaccine treatment before each cycle	<2,000 IU/mL	<1,500 IU/mL	68 weeks	46.67%/40.74%	(43)

PEG-IFN-α, pegylated interferon-α; NAs, nucleos(t)ide analogs; ETV, entecavir; HBsAg, hepatitis B surface antigen; HBeAg, hepatitis B e antigen; IHC, inactive HBsAg carrier; GM-CSF, granulocyte-macrophage colony stimulating factor.

Long-term HBV suppression by TDF could lead to the regression of fibrosis and cirrhosis (46). Papatheodoridis et al. retrospectively analyzed 147 HBeAg-negative CHB patients with or without IFN-*α* treatment and found that fibrosis regression rate in patients underwent IFN-*α* therapy was remarkably higher than that in untreated patients (17.5% *vs* 4%) according to the histological assessments, and the effect of fibrosis regression was mainly observed in patients who achieved sustained biochemical responses (47). Buster et al. also revealed that PEG-IFN-*α*-2b induced higher rate of HBeAg seroconversion, HBV DNA inhibition, and improvement of liver fibrosis in HBeAg-positive CHB patients with advanced fibrosis (26). However, Chen et al. showed that fibrosis regression rate was similar between ETV monotherapy (68%, 32/47) and PEG-IFN-α-2a add-on (56%, 60/108) in CHB patients with pre-treatment biopsy-proven Ishak fibrosis score 2, 3, or 4 after 78 weeks of therapy (27). In consistent with the findings by Chen et al. (27), our present data also indicated that 48-week PEG-IFN-*α*-2b might induce neither regression nor progression of liver cirrhosis based on Fibroscan and sonography assessments, which presented the comparable LSM, thickness and length of spleen, and diameter of portal vein between baseline and 48 weeks post-PEG-IFN-*α*-2b-based therapy regardless of monotherapy or add-on strategy in patients with HBV-related compensated liver cirrhosis. The ALB level also maintained stable during the observational period. In several patients, we also performed CT or MRI scans 48 weeks post therapy. The degree of liver cirrhosis did not present significantly changes in response to PEG-IFN-*α*-2b treatment. Further histological assessments should be performed to confirm the results.

We observed good safety profiles in HBV-related compensated liver cirrhosis in response to PEG-IFN-*α*-2b therapy. No unexpected severe AEs were reported. A 38-year old male patient suffered with grade 1 ascites 40 weeks post PEG-IFN-α-2b add-on ongoing TAF treatment. The patient felt mild abdominal distension, and received sonography assessment for a 1.3 cm depth of liquid dark area. This indicated grade 1 and a small amount of ascites. We also re-analyzed the CT scan of the patient in the baseline, and found a small amount of fluid accumulation around the spleen. Although the aminotransferase levels of this patient maintained stable during treatment, we still discontinued PEG-IFN-*α*-2b therapy and continued TAF therapy. The ascites disappeared 4 weeks post PEG-IFN-α-2b withdrawal. The liver function remained stable and no decompensated symptoms were reported during the following-up period. It was also well accepted that hepatic decompensation and HCC progression was uncommon but not eliminated even in patients with HBV-related cirrhosis receiving antiviral therapy (48, 49). Thus, it was still could not confirm the association between PEG-IFN-*α*-2b and hepatic decompensation in this patient. We also found the ALT elevation induced by PEG-IFN-α-2b, which might reflect immune clearance of HBV. However, ALT elevation more than five times of ULN was only found in four patients and no patients discontinued PEG-IFN-*α*-2b therapy due to ALT flare. Moreover, the down-regulations of WBC, neutrophils, platelets, and HGB levels were found during the treatment period. It was interesting that the recovery of netrophils and platelets count seemed faster in PEG-IFN-α-2b add-on strategy, indicating PEG-IFN-α-2b plus NAs treatment might have slighter affection to blood routine test.

There were several limitations in the current study. Firstly, we only enrolled 54 patients with HBV-related compensated cirrhosis in a single center. Large scale, multi-center cohort study should be performed to confirm the present findings. The histological assessments for liver cirrhosis before and after PEG-IFN-α-2b therapy should also be investigated. Secondly, we only observed the 48-weeks of PEG-IFN-α-2b treatment for enrolled patients. For liver cirrhosis patients, longer follow-up would be valuable to assess the sustainability of the virological response and whether the liver function improvements are maintained. The off therapy response and HCC progression should also be monitored for a long-term follow-up period. Thus, the long-term studies and innovative combination therapies should be performed in further research.

## Conclusion

In summary, a 48-week finite course of PEG-IFN-α-2b therapy was well-tolerated, and reduced HBsAg level without accelerating disease progression in patients with HBV-related compensated cirrhosis. Our findings might help inform optimal therapeutic strategy for HBV-related compensated cirrhosis, leading to fewer incidences of end-stage liver diseases. Importantly, integrating personalized approaches is pivotal for PEG-IFN-α-2b-based therapy in clinical practice, which will provide a comprehensive and forward-looking perspective during the treatment of HBV-related cirrhosis.

## Data Availability

The raw data supporting the conclusions of this article will be made available by the authors, without undue reservation.
